# Insight into Rheological Properties and Structure of Native Waxy Starches: Cluster Analysis Grouping

**DOI:** 10.3390/molecules29112669

**Published:** 2024-06-05

**Authors:** Jacek Lewandowicz, Joanna Le Thanh-Blicharz, Artur Szwengiel

**Affiliations:** 1 Department of Food Concentrates and Starch Products, Prof. Wacław Dąbrowski Institute of Agriculture and Food Biotechnology—State Research Institute, Starołęcka 40, 61-361 Poznan, Poland; joanna.lethanh-blicharz@ibprs.pl; 2 Department of Food Technology of Plant Origin, Faculty of Food Science and Nutrition, Poznań University of Life Sciences, Wojska Polskiego 31, 60-624 Poznan, Poland; artur.szwengiel@up.poznan.pl

**Keywords:** potato starch, corn starch, rice starch, texture, amylose, SEC, GPC, HCA, PCA

## Abstract

Recent interest in the use of waxy starches in food production is due to the possibility of replacing chemically modified starches as texture-forming agents with native starch analogues. However, there is a lack of a coherent research comparing different varieties of commercially available waxy starches with respect to their molecular and functional properties. Therefore, the objective of this study was to compare native waxy starches from potatoes, corn, and rice, with particular attention to rheological characteristics in relation to molecular structure. The investigated potato, corn, and rice starch preparations were characterized by significantly different molecular properties due to both botanical origin of starch and variety. The molecular weights of waxy starches were significantly higher than those of their normal counterparts. This phenomenon was accompanied by a more loose conformation of the waxy starch macromolecule in solution. The presence of amylose confers the ability to coagulate starch sol into gel, resulting in substantial changes in the rheological properties of starch paste, and waxy starch pastes being characterized by more viscous flow and smoother texture. Hierarchical cluster analysis indicated that differences between functional properties are more notable for normal than for waxy preparations, in which potato starch, regardless of its variety, was characterized by the most unique characteristics.

## 1. Introduction

Starch is a plant polysaccharide composed exclusively of D-glucose monomers linked by α-glycosidic bonds. It is made up of two fractions, essentially unbranched amylose and branched amylopectin. Amylose consists of relatively long linear anhydroglucose chains, about 99% of which are connected with α-1,4-glycosidic bonds and up to 1% with branching α-1,6-glycosidic links. Its molecular mass ranges from 1 × 10^5^ to 1 × 10^6^ g/mol. Amylopectin is characterized by a much higher molecular mass of about 1 × 10^7^–1 × 10^9^ g/mol and its branched structure is the result of a much larger number of α-1,6-glycosidic bonds, which is estimated to be approximately 5% [1,2,3].

Depending on the botanical origin, amylose usually constitutes 10–35% of starch. Specific values also depend on the method used for their determination. There are also specific plant varieties whose starch contains an unusually high proportion of amylose or amylopectin. High amylose starches contain up to 70% of amylose, while waxy starches consist almost entirely of amylopectin [3,4,5]. The ratio of amylose to amylopectin affects the functional properties and applicability of starch. High amylose starches are particularly useful in the production of starch preparations resistant to amylolytic enzymes. However, their application in food technology is limited. On the other hand, high amylopectin (waxy) starches, compared to ordinary varieties, are characterized by a number of unique features, especially useful in food processing [3,6,7,8,9].

For economic reasons, most of the starch produced in the world comes from corn, wheat, and potatoes, and in small amounts (less than 5% in total), it is obtained from other plant materials. The production of tapioca, that is, starch obtained from cassava, is also important on a global scale [1,10]. Rice starch is also of particular importance due to its unique physicochemical properties and the popularity of rice cultivation in Asia. On a regional scale, starch is also obtained from peas, millet, barley, potato wolf tubers (sweet potatoes), reed arrowroot rhizomes, saga palm core, unripe banana fruit, and patch-shaped root (kudzu). In times of scarcity, starch has also been obtained from rye and oats [11,12].

The first botanical variety of a plant reported to produce only amylopectin was maize, which was named waxy due to the hard waxy texture of the endosperm [13], and 13 years later an attempt was made to characterize its properties [14]. A year earlier, Parnell [15] described the presence of amylopectin in sticky rice, which has probably been cultivated for more than 2000 years [16]. Among the waxy starches, the one derived from corn is of the greatest economic importance [17]. Commercially available waxy rice starch is more expensive, so its economic importance is definitely smaller. Wild varieties containing waxy starch are also found among other cereals: barley, sorghum, amaranth, and millet. However, for economic reasons, starch from these plants is not obtained on an industrial scale. As waxy starches from other plant species were expected to have new beneficial properties, waxy varieties of wheat, potatoes, and tapioca were developed in the 1990s [18,19,20,21]. Since then, intensive studies have been carried out to expand the range of application of waxy starches for both food and non-food purposes. Several physical and chemical modification processes, including nanoparticle formation, are also under development [22]. The interest in waxy starches in food production results from the trend of the so-called clean label and the potential possibility of replacing chemically modified starches as texture-forming agents with natural analogues [23]. Waxy starches have a certain set of features that make them similar in terms of properties and structure. For example, all waxy starches reveal a high type of pasting characteristics, with a rapid increase in viscosity within a narrow temperature range and presence of a viscosity peak, while normal cereal counterparts reveal a medium type, with a progressive increase in viscosity over a wide temperature range and the lack of a viscosity peak [18,24]. However, there are a number of detailed differences between individual starches, which creates the need for further research to expand the possibilities of their use in industrial practice.

The functionality of starches is primarily defined by their rheological properties. However, they can be defined using different methods and different apparatuses. Viscographs (including Rapid Visco Analysers), which enable measurements of gelatinization temperature and other parameters of technological importance, are commonly used. Rheometers that allow for rheological analyses at a precisely defined shear rate are particularly useful. There is also an opportunity to perform nondestructive rheological analyses of gels thanks to the use of oscillatory rheometers [25]. The functional properties of starch preparations are immanently related to their structure as they reflect polymer–solvent interactions, i.e., starch–water–solute interactions [26]. One of the methods to analyze the structure of starch in relation to its functionality is size exclusion chromatography (SEC) with triple detection. This employs a concentration detector, a viscometer, and light scattering detectors and thus provides the possibility of measuring not only the molecular mass distribution, but also the parameters of macromolecules in solution, i.e., the radius of gyration and the hydrodynamic radius [27,28,29,30].

The most common commercially available starches, such as corn, wheat, potato, cassava, or rice, have been thoroughly examined in terms of their rheological properties and molecular structure [25]. Similarly, waxy corn has been thoroughly characterized and fills a specific market niche, contrary to waxy potato starch, which was introduced to the food market quite recently [22,31]. Moreover, there is still a lack of coherent basic study comparing all different varieties of commercially available waxy starches. Therefore, the aim of this study was to compare native waxy starches from potatoes, maize, and rice, with particular attention to rheological characteristics in relation to their molecular structure.

## 2. Results and Discussion

### 2.1. Proximate Composition of Starch Granules

The functional properties of starch are mainly influenced by the ratio of amylose to amylopectin; however, microconstituents alter these properties significantly [32,33]. The content of both of these polyglucans is determined by biosynthesis of the starch granule, while the microconstituents are mostly determined by the technology of the isolation process. The composition of starch granules is presented in Table 1.

Theoretically, the amylose content of waxy starches should be equal to 0%. However, due to the inaccuracy and multiplicity of methods used for determination of amylose [3,34], the values reported in the literature range from zero to a few percent, depending on the botanical origin of starch and the procedure used [20,35,36,37,38,39,40,41]. The amylose content of investigated waxy starches ranged from 2.5 to 3.0%, but the observed differences were statistically insignificant. The amylose content of normal starches was within average values reported in the literature, i.e., 18.3–31.0%, 22.4–32.5%, and 4–29% for potato, corn, and rice starch, respectively [32,35,36,37,38,40].

Starches isolated from tubers and roots are significantly different from cereal ones in terms of impurities (lipid and protein fractions) [32]. Starches isolated from potatoes and pulses are considered as free of lipids, while cereal starches tend to form amylose–lipid complexes that can be removed neither in the isolation process nor by extraction with organic solvents (without disrupting starch granular form). The protein content in starch isolated on an industrial scale ranges between 0.1–0.7% [2]. All investigated starches were within this range, with the exception of waxy rice preparation. The slightly higher protein content of this preparation is typical as a result of the association of starch granules with rice protein [32].

Depending on the botanical origin, starch can be constituted up to 1% of minerals [42], but these values generally do not exceed 0.3% [32]. The highest ash content was recorded for both potato starch varieties and normal rice starch. The increase in the amount of ash present in potato starch is associated with the presence of phosphate groups, which have the ability to bind metal ions through ionic bonds [35,42]. This phenomenon explains the slightly lower value of the ash content observed for the waxy potato variety, which can be associated with a lower phosphorus content. The lowest ash content was observed for both corn varieties, which is consistent with the data from the literature [32,35].

Almost all starch varieties contain a certain amount of phosphorus, which can be present in the form of phospholipids or bound as esters of orthophosphoric acid [2]. Cereal starches contain between 0.01–0.07% phosphorus mainly in the form of phospholipids. Potato starch can contain up to 0.09% phosphorus which is primarily bound to the C-6 carbon of amylopectin as an ester of orthophosphoric acid [32]. Although the amount of phosphorus present in the starch granule may seem insignificant, it alters pasting characteristics when bound as a phosphate monoester [43]. The highest phosphorus content was observed for normal potato starch, followed by waxy potato starch. Both values were within the range reported in the literature by various researchers [44]. Various researchers also observed a slightly lower phosphorus content in waxy potato starch [36,37], which may seem strange considering the binding mechanism of phosphorus in potato starch by amylopectin. The phosphorus content in cereal starches was significantly lower, and as in the case of potato starch, waxy varieties were characterized by lower values.

### 2.2. Paste Clarity

The clarity of starch paste is an important quality parameter that influences the field of its application and determines the assortment of products in which it can be used [45]. The paste clarity is influenced by numerous factors including the ratio of macroconstituents, amount of microconstituents, and molecular structure [2,45,46]. The pastes obtained from potato starches were characterized by significantly higher clarity than those of cereals (Table 1). Furthermore, waxy starch pastes were characterized by a higher clarity than their normal counterparts. This phenomenon was especially evident for cereal starches. The observed differences can be directly related to the swelling characteristics of the starch preparations (Section 2.5). Starch granules that are more easily swollen leave fewer granule remnants, and thus, the starch paste is less opaque. Moreover, the clarity of potato starch is further enhanced by the presence of phosphorus bound in the form of monoester, which prevents the association of starch molecules that normally lead to a decrease in transmittance of the starch paste [45].

### 2.3. Starch Granule Size and Morphology

The size and morphology of the starch granules show great diversity based on the botanical origin of the material used for their isolation [37,47]. The average size of the starch granule can range from sub-micron levels up to 100 μm. Based on average size, starch granules can be classified as large >25 μm, medium 25–10 μm, small 10–5 μm, and very small <5 μm [48]. Furthermore, according to their shape, granules can be classified as spherical, oval, disk, polygonal, elongated, and kidney [49].

**Figure 1:**
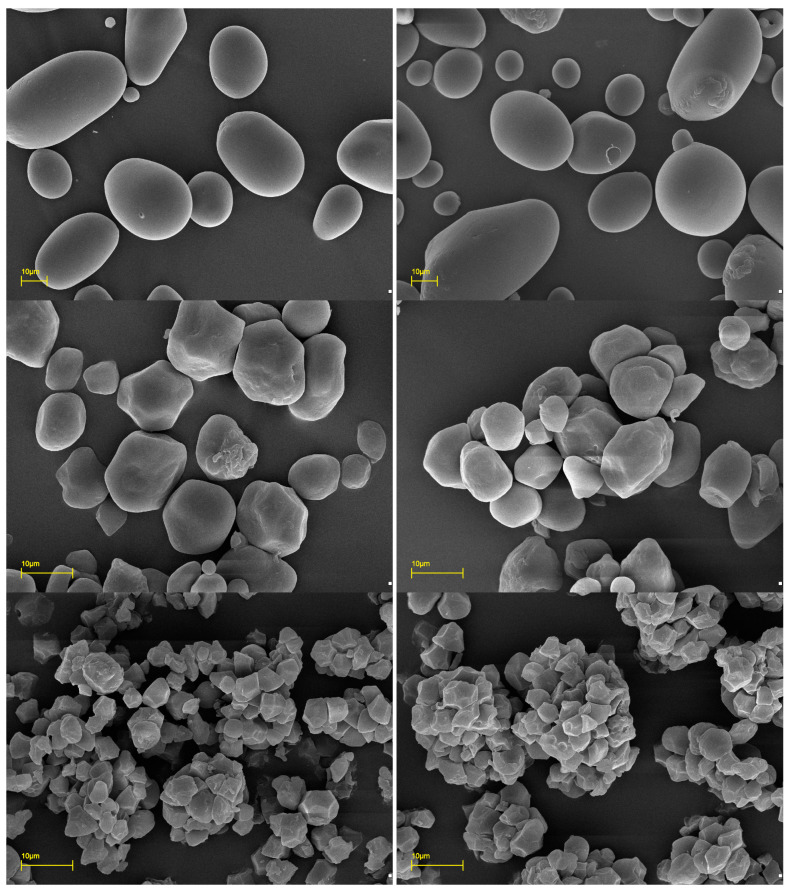
Scanning electron microphotographs of native starch granules, from top to bottom: potato, corn, and rice, respectively; from left to right: normal and waxy varieties, respectively.

Scanning electron microphotographs are presented in Figure 1, whereas the distribution of starch granule size is presented in Table 2. The first decile (D_1_) and last (D_9_) represent the smallest and the largest 10% of the granules in the starch preparation. The fifth decile (D_5_) represents the median size of starch granule.

Potato starches were characterized with similar granule size and morphology, i.e., medium and large size granules with a spherical or oval shape. Similarly, both rice starch preparations were characterized by similar properties to each other, consisting of very small, polygonal granules. It should also be noted that rice starch granules tend to form agglomerates. This phenomenon is related to the atypical biosynthesis of rice starch that occurs simultaneously for numerous granules in one amyloplast [2]. This results in agglomeration that can be visible in SEM photographs with clumps larger than 30 μm in diameter.

Corn starches were the only preparations that revealed differences due to the botanical variety. Although both preparations were characterized by similar morphology, i.e., polygonal and spherical granules, their median size was significantly different. Normal corn starch had primary granules that should be classified as medium in size, while the last quartile of waxy corn starch consisted of very large granules.

Considering food technology, observed differences between starch granule size and morphology are of minor importance, as most starch preparations are utilized in gelatinized form. Nevertheless, in certain applications, i.e., when used in suspension (cardboard glue) or in granular form (cosmetic powder), observed differences may be crucial quality determinants.

### 2.4. Size Exclusion Chromatography

**Figure 2:**
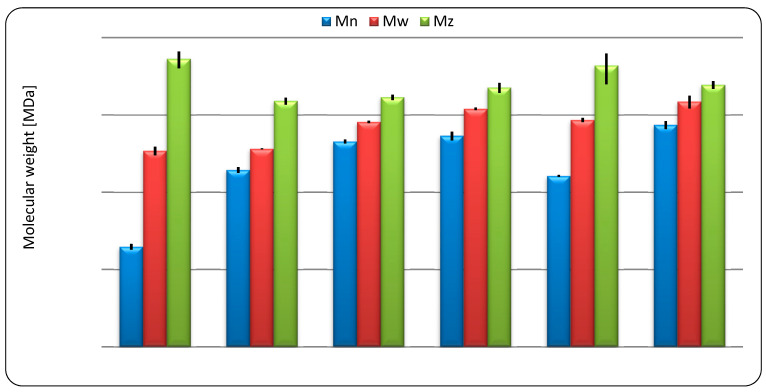
Molecular weight distribution of native starches.

The molecular weight and conformation of starch macromolecules in solution determines the rheological properties of starch pastes. The molecular mass distribution of the investigated preparations is presented in Figure 2, whereas the hydrodynamic parameters are listed in Table 3. The molecular weight distribution was similar for all waxy preparations as well as normal corn starch. A comparable degree of polymerization of waxy cereal starches was previously reported [50]. All of these preparations were also characterized by a similarly low polydispersity index (M_w_/M_n_). However, it should be noted that the highest values of number (M_n_) and weight (M_w_) average molecular weights were recorded for the waxy rice preparation and equaled 74.5 and 149.2 MDa, respectively. The highest value of Z-average molecular weight (M_z_) was observed for normal potato starch, which surprisingly was also characterized by the lowest values of M_n_ and M_w_—1.9 and 34.4 MDa, respectively. This phenomenon is related to the high polydispersity of this starch. Similar characteristics of the molecular weight distribution can also be observed for normal rice starch, which had relatively low M_n_ and high M_z_, as well as the M_w_/M_n_ ratio.

Regardless of botanical origin, the waxy starch variants were those of higher M_n_ and M_w_ than their normal counterparts; this is related to the fact that amylopectin is a starch fraction of higher molecular weight. Jiranuntakul and coauthors obtained similar results [51], indicating that the elution time for amylopectin is similar for normal and waxy preparations of potato, corn, and rice starch, and the difference arises from the second peak, which is attributed to the presence of amylose. However, this phenomenon was not observed for M_z_, which, apart from potato starch varieties, did not differ significantly (between all investigated samples). The effect of constituting purely of amylopectin was clearly observed for waxy starches in parameters that describe the shape of the starch macromolecule in solution—hydrodynamic radius (R_h_) and radius of gyration (R_g_). Normal starches were characterized by lower values of R_h_ and higher values of R_g_. This resulted in the ratio of these parameters reaching values above 2 for normal starch preparations, indicating that they adopt a rod-like shape in solution, whereas waxy preparations adopt a more packed structure of statistical coil [52]. The rest of the investigated parameters did not differ significantly within the same botanical origin, with the exception of corn starch. This indicates that although the molecular mass distribution of normal and waxy corn starch is similar, their molecular structures are significantly different. This is also consistent with the structure of high-amylose corn starch preparations, which is fairly different from normal corn [8].

### 2.5. Rheological Characteristics

**Figure 3:**
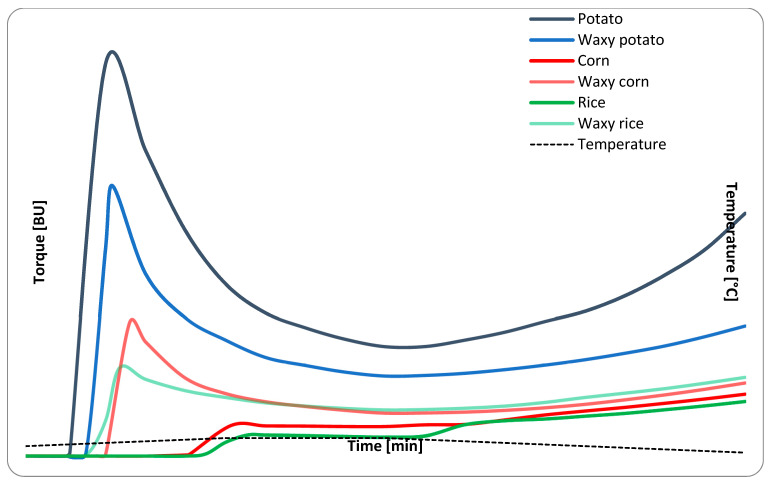
Pasting profile of 5% native starch suspensions.

Pasting characteristics form one of the key parameters that determine the possible range of applications of starch in food technology [53]. Typically, three types of swelling behavior are distinguished, i.e., high, medium, and restricted. The first one is characterized by a sharp viscosity peak and pronounced breakdown followed by a significant setback. The latter two are characterized by a progressive increase of viscosity throughout all phases, with the difference observed in stabilization during the holding period for the medium types [30]. Tuber starches as well as waxy starches, regardless of botanical origin, are characterized by a high type of swelling characteristics. On the other hand, cereal starches reveal a medium type of swelling characteristics [32]. The investigated products did not deviate from that pattern (Figure 3). Rapid gelatinization (as indicated by the viscosity peak) of waxy preparations is the result of a lack of amylose that inhibits granule swelling [31].

The final viscosity is the most important parameter in determining the performance of starch as a thickener. The highest values of this parameter were recorded for normal and waxy potato starch, respectively. Significantly lower values were recorded for the remaining analyzed preparations that ranged between 262 and 373 BU. Potato starches were also characterized by the lowest gelatinization temperatures, of 62 °C and 65 °C for the normal and waxy variety, respectively, followed by cereal waxy preparations reaching 65 °C for rice and 69 °C for corn. Normal cereal starches had gelatinization temperatures at least 15 °C higher than their waxy counterparts. Similar but less pronounced differences between pasting temperatures of normal and waxy cereal starches were also reported in the DSC analysis [31].

Secondary pasting parameters, i.e., breakdown and setback, were highly influenced by type of pasting characteristics, and their direct comparison between different swelling patterns would be inaccurate. All waxy and normal potato starch pastes were characterized by a pronounced breakdown that lasted until the end of the holding period. The extent of breakdown was related to the peak viscosity of the sample. The breakdown value of cereal starches was below 10 BU, which is also below the error of the method. The setback value for all cereal starches (including waxy) ranged between 140 and 165 BU, and observed differences can be considered as statistically insignificant (*p* > 0.05). The increase in viscosity for potato preparations was higher, which was especially evident for the normal variety. This was the result of a rapid increase in viscosity as the paste reached a temperature level below 50 °C, and thus can be linked to the gelling phenomenon.

Among the most commonly performed rheological trials are flow curves, which allow for basic characterization of the sample in terms of viscosity and resistance to shear forces. Starch pastes are non-Newtonian, pseudoplastic fluids with thixotropic properties, and their flow is often described by the Ostwald–de Waele model [30]. The parameters of this equation for the upward and downward curves, as well as the area of hysteresis loop of thixotropy, are presented in Table 4. The employed model was very well fitted to the experimental data as indicated by the values of the coefficient of determination exceeding 0.95 by a large margin. The consistency index increased in the order: rice < corn < potato starch paste, whereas waxy starch preparations had significantly lower K values. The only exception was observed for the waxy rice starch paste; nevertheless, a similar pattern of the results was observed for the final viscosity during analysis of pasting profile. Relatively high values of consistency coefficients recorded for normal starches may be related to gelling characteristics of these starch products, as amylose is the crucial component responsible for this phenomenon. This theory is also supported by the fact that K values for the downward curve were reduced at least twice for normal preparations, whereas for waxy ones the reduction was at most by one third. This indicates the destruction of the thixotropic gel structure in normal starch pastes. The flow index *n*, which is a measure of convergence with Newtonian flow, has increased in the following order: normal corn < both rice < normal potato < waxy potato and corn starch. During the second cycle, when the shear rate decreased, the *n* value of normal starch pastes dropped drastically, whereas little to no effect of applied shear forces was observed for waxy preparations. This type of flow characteristic can be once again attributed to amylose, which creates a thixotropic structure, resulting in poorer rheological stability of normal starch pastes. This observation is also supported by the calculated thixotropy value. The only exception was observed for waxy rice starch, which had the highest value of thixotropy among cereal starches. This phenomenon may be partially related to the fact that its macromolecule had the largest hydrodynamic radius.

The determination of rheological properties in controlled deformation enables characterization of paste properties without destroying the thixotropic structure. Mechanical spectra in linear viscoelastic range obtained in that manner can also be fitted to the power law equation. Table 5 represents data for storage G′ and loss G″ modulus, which are also referred to as elastic and plastic, respectively. This is related to the fact that G′ describes the energy that was temporally stored in the sample (and can be recovered after the shear forces have ceased), while G″ represents the energy used for the initiation of the flow (and which cannot be recovered) [54]. All starch pastes analyzed were characterized by G′ > G″ in considered angular velocity range; nevertheless, for normal starch preparations the storage modulus was by order of magnitude higher than loss modulus. This observation is supported by the base values of the power law equation, K′ and K″, presented in Table 5, whereas for waxy starch preparations, K′ and K″ were within the same order of magnitude. This indicates that normal starch pastes should be considered as gels, whereas waxy starch pastes do not meet the phenomenological definition of the term gel and should be referred to as sols [55]. Significant differences in mechanical properties of normal and waxy starch pastes were also related to the slope of the mechanical spectra obtained. Normal starch pastes were characterized by almost plateau-like curves indicated by values of *n*′ close to 0, while for waxy pastes this parameter was several times higher. A similar, but less evident relationship was observed for the *n*″ exponent. Lastly, significant differences between starch pastes of waxy and normal varieties were manifested by mean values of phase angle tangent δ, which were lower in the case of pastes with jelly-like structure. The above observations confirm the crucial role of amylopectin in the formation of the gel network in starch pastes.

### 2.6. TPA

Texture is a multiparameter sensory attribute of a food product; nevertheless, several instrumental methods of determination of texture can be used. Among them is the profiling method of texture description (TPA), which is applicable both for sensory and instrumental measurements [56]. A universal texture profile of investigated starch pastes is presented in Table 6. The profiles obtained were typical for starch pastes, i.e., parameters that differentiated analyzed samples were hardness, adhesiveness, and gumminess [25,30]. These types of texture characteristics should be linked with liquid-like behavior that results in springiness values close to unity. At the same time, the cohesiveness values are usually considerably lower because of the thixotropic characteristics of starch paste. The hardness values of normal starch pastes were significantly higher than those of their waxy counterparts, which can be attributed to the gelling characteristics of normal starch. The only exception was observed for rice starch pastes, in which case observed differences were insignificant; this is the result of the relatively low viscosity of normal rice starch paste. Similar conclusions can be drawn on the basis of the adhesiveness results, which in the case of all waxy as well as normal rice pastes were in fact inexistent. Lastly, gumminess was also the highest for normal corn and potato starch paste and statistically indifferent for the rest of the samples. This indicates that normal starch pastes have a relatively stiff texture, whereas waxy pastes have a smoother one. This phenomenon is partially influenced by the viscosity of the product and therefore can plausibly be concentration-dependent.

### 2.7. Statistical Analysis

**Figure 4:**
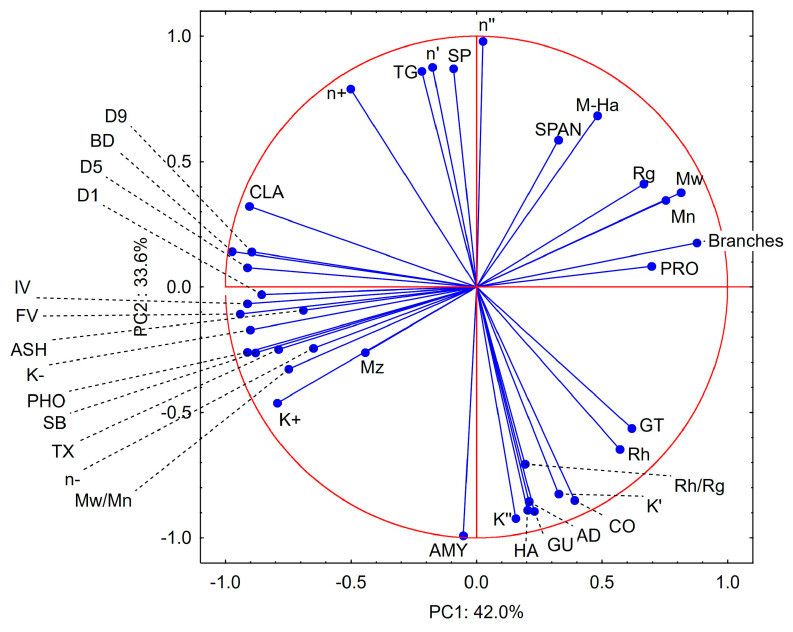
PCA loadings plot for data presented in Table 1, Table 2, Table 3, Table 4, Table 5 and Table 6 and gelatinization parameters from Figure 3. Explanatory notes: AMY—amylose content, PRO—protein content, ASH—ash content, PHO—phosphorus, CLA—paste clarity, D1—granule size (first decile), D5—granule size (median), D9—granule size (last decile), SPAN—granule size distribution, M_n_—number average molecular weight, M_w_—weight average molecular weight, M_z_—Z-number average molecular weight, M_w_/M_n_—polydispersity index, IV—intrinsic viscosity, R_h_—hydrodynamic radius, R_g_—radius of gyration, M-Ha—Mark–Houwink equation α parameter, Branches—number of branches per molecule, GT—gelatinization temperature, FV—vfinal viscosity, BD—breakdown, SB—setback, K+—consistency index (upwards curve), K−—consistency index (downwards curve), *n*+—flow behavior index (upwards curve), *n*−—flow behavior index (downwards curve), K′—base value of power law equation (storage modulus), K″—base value of power law equation (loss modulus), *n*′—factor of power law equation (storage modulus), *n*″—factor of values of power law (loss modulus), TG—average value of phase angle tangent, HA—hardness, AD—adhesiveness, CO—cohesiveness, SP—springiness, GU—gumminess.

**Figure 5:**
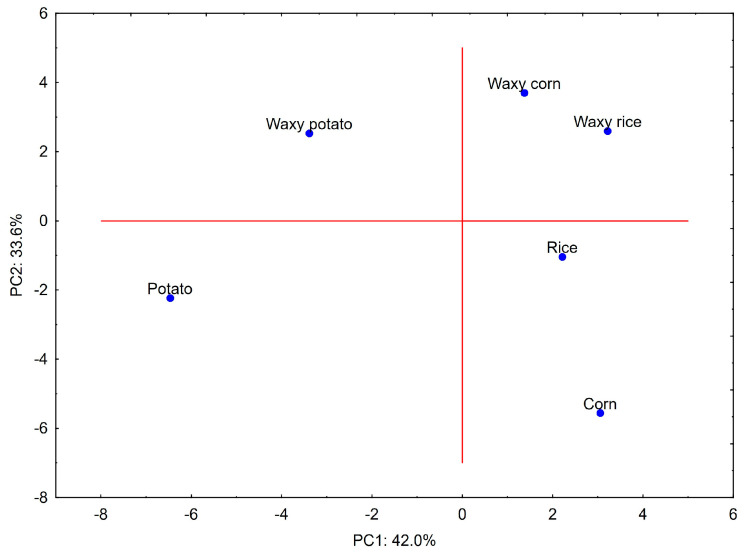
PCA score plot for native starches.

In order to explore the relationship between investigated rheological parameters, structure and proximate composition of starch, principal component analysis (PCA) was performed. The first two principal components presented on the PCA plots explain more than 75% of the total variance (Figure 4 and Figure 5). The variables presented on the score plot can be categorized into four major groups, which align almost perfectly within the boundaries of the quadrants of the coordinate system. The first group, with PC1 loading values close to 0 and PC2 close to 1, mostly includes parameters that are related to slope of mechanical spectra and average value of phase angle tangent. The second group, with PC1 loadings slightly above 0 and PC2 close to 1, includes parameters related to molecular mass distribution, branching, and hydrodynamic radius. The correlation among these parameters is obvious and is related to the fact that branched starch molecules have a larger molecule size and higher molecular mass. The third group of parameters is considerably larger than two previous ones and is characterized by PC1 close to 0 and PC2 close to −1. This group links the amylose content with solid texture, i.e., high TPA parameters and K′ and K″ equation constants. This group of variables was also negatively correlated with the first group, which described parameters associated with more viscous paste characteristics. These observations were confirmed by Pearson’s correlation coefficient values greater than 0.8 (*p* < 0.05) for amylose and the rest of the parameters from group 1 and 3 (except K′). The fourth group with negative values of both PC1 and PC2 is the largest and links viscosity during gelatinization with phosphorus and ash content, as well as flow characteristics. The link with pasting parameters and phosphorus content was also proven by Pearson r > 0.8 (*p* < 0.05).

The PCA score plot highlighted the differences between waxy and normal, as well as between cereal and tuber starches. The first principal component differentiated the waxy and normal preparations. Positive PC1 values were observed for waxy preparations and may be linked with viscous behavior of starch pastes. Conversely, normal preparations had negative values of PC1, which should be linked to their partially elastic behavior. The second principal component differentiated cereal and tuber starches, with positive PC2 values for the first and negative for the latter. This differentiation may be related to the higher viscosity of tuber starches and more compact size of starch macromolecules in solution accompanied by lower branching.

**Figure 6:**
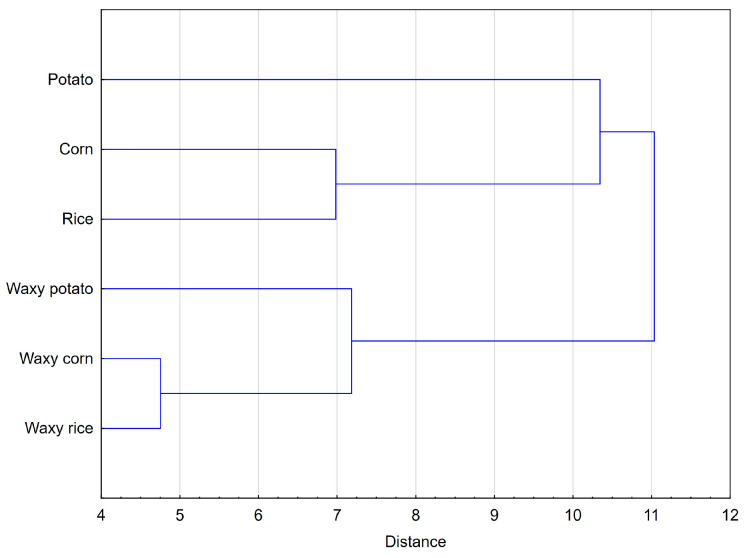
Hierarchical cluster analysis of data representing proximate composition, molecular and rheological properties, as well as texture of native starch.

The hierarchical cluster analysis (Figure 6) confirmed the initial classification of starch products prepared on the basis of PCA score plot, as indicated by two main clusters formed separately by normal and waxy starches. The calculated cophenetic correlation coefficient (CPCC) for obtained clustering was 0.66 and can be considered satisfactory. The waxy starch cluster included two products that were the most similar, which were waxy cereal starches. The second pair that was most similar was formed by their normal counterparts. Potato starches stood out from the rest in their clusters due to their unique rheological properties that arise from a substantially different molecular structure.

## 3. Materials and Methods

### 3.1. Starch

Commercially available native starch of both normal and waxy variety obtained from potato, corn, and rice were the studied material. The starch was manufactured by: PPZ Trzemeszno (Trzemeszno, Poland)—normal potato variety; Avebe (Veendam, The Netherlands)—waxy variety; Agrana Starke GmbH (Aschach an der Donau, Austria)—both corn varieties; and Beneo (Leuven, Belgium)—both rice varieties. All analyses were performed on starch dry matter basis, determined by oven drying at 105 °C for 3 h. Starch samples were stored in two double-seal, resealable polypropylene bags (50 μm) in order to minimize changes in moisture content. The determined moisture content was, respectively, 18.48%, 18.50%, 12.43%, 12.36%, 10.42%, and 12.38% for potato, waxy potato, corn, waxy corn, rice, and waxy rice starch.

### 3.2. Proximate Analysis of Starch

Amylose content was determined using K-AMYL assay kit (Megazyme, Wicklow, Ireland). The kit procedure is based on precipitation of amylopectin by forming a complex with concanavalin A, described by Morrison and Laignelet [57]. The samples were degreased according to the modification proposed by Yun and Matheson [58]. The spectrophotometric determination was carried out on double-beam spectrophotometer Cary 5E (Varian, Mulgrave, Australia).

Protein content was determined according to PN-EN ISO 3188:2000 [59]. The mineralization procedure was performed using K-424 digestion unit equipped with B-414 acid scrubber (Büchi, Flawil, Switzerland). Distillation process was carried out with the assistance of B-324 distillation unit. The distillate was titrated with the solution of 0.1 n hydrochloric acid against phenolphthalein.

Ash content was determined according to PN-EN ISO 3593:2000 [60]. Starch samples of approximately 5 g were incinerated at 900 °C using muffle furnace (Czylok, Jastrzębie-Zdrój, Poland).

Phosphorus content was determined according to procedure proposed by Hogen [61] using atomic absorption spectroscopy with electrothermal atomization using SpectrAA 800 spectrophotometer equipped with GTA-100 graphite furnace (Varian, Mulgrave, Australia). Samples were mineralized using nitric (V) acid using MDS-2000 (CEM, Charlotte, NC, USA). The measurement was performed with default setting at λ = 213.6 nm with deuterium lamp background correction. Nickel ions at a concentration of 0.1% were used as matrix modifiers.

### 3.3. Paste Clasity

Paste clarity was determined according to the modified procedure described by Craig et al. [45]. Transmittance (%T) of starch paste prepared with assistance of Brabender viscograph in concentration of 1% (Section 2.7) was measured immediately after preparation at λ = 650 nm using Cary 5E spectrophotometer (Varian, Mulgrave, Australia).

### 3.4. Scaning Electron Microscospy

Scanning electron microphotographs were taken with the assistance of an Evo 40 series microscope (Carl Zeiss, Jena, Germany) operating at 17 kV accelerating voltage. Samples were coated with gold using an SCD 050 sputter coater (Oerlikon Balzers, Liechtenstein).

### 3.5. Size of Granules

Starch granule size distribution was determined using laser particle sizer Analysette 22 NanoTec plus equipped with wet dispersion unit (Fritsch, Idar-Oberstein, Germany). The measurement of rice starch samples was preceded by sonication at 36 kHz for 2 min in order to separate agglomerates, typical for rice starch preparations.

### 3.6. Size Exclusion Chromatography

Molecular mass distribution and hydrodynamic parameters of starch were determined using high-performance size exclusion chromatography (SEC) with triple detection OMNISEC system (Malvern Instruments, Malvern, Great Britain) equipped with Viscotec TDA 305 multi-detector. The sample was dissolved in dimethyl sulfoxide:water binary mixture (9:1) to obtain concentration of 1.5 mg/mL. Sample preparation and separation procedure were described in detail previously [30]. The calculations were performed using the OmiSEC 4.7. software (Malvern, TX, USA). Calculation principles of the average molecular masses (Mn, Mw, Mz), intrinsic viscosity (IV), hydrodynamic radius (Rh), radius of gyration (Rg), and number of branches per molecule (Bn) were described previously by Szwengiel and co-authors [62].

### 3.7. Pasting Characteristics

Pasting characteristics of 5% starch aqueous samples were recorded with a Brabender Viscograph (Duisburg, Germany), using the following conditions: measuring cartridge 0.07 Nm, heating and cooling rate of 1.5 °C/min, temperature range 25–92.5–25 °C, holding period of 20 min. Obtained pasting curves were analyzed in terms of gelatinization temperature °C, peak viscosity BU (Brabender units), breakdown BU, setback BU, and final viscosity BU. All analyses were performed in duplicate.

### 3.8. Rheological Measurements

Rheological properties of starch pastes obtained at stage 2.7 were determined using a RheoStress1 rheometer (Haake Technik GmbH, Vreden, Germany) equipped with a DC30- W19 temperature control unit. Before the measurement, samples were set to equilibrate in measurement system at 20 °C for 5 min. Data collection and calculations were made using RheoWin 3.61 software.

#### 3.8.1. Rotational Rheometry

Rotational rheometry was based on Z20 DIN Ti coaxial measurement geometry. Flow curves were determined within 0.1–600 s^−1^ shear rate range in time of 2 min. Obtained flow curves were described with the Ostwald–de Waele equation [30]. Moreover, thixotropy, understood as area of hysteresis loop formed between upward and downward flow curve, was calculated. Measurements were performed immediately after preparation of starch pastes.

#### 3.8.2. Oscillatory Rheometry

Mechanical properties were determined using 60 mm parallel plate measurement system (PP60). Measurements were performed within linear viscoelastic region 0.1–10 rad/s angular velocity at an amplitude of 0.05% and gap of 1.0 mm. Obtained storage (G′) and loss (G″) modulus data were fitted to power law equation. Phase angle tangent (Tg δ) was expressed as an average value [63]. Mechanical properties were determined on pastes stored for 24 h at room temperature (20–25 °C).

### 3.9. Universal Texure Profile

Universal texture profiles were determined with assistance of TA-XT2 texturometer (Stable Micro Systems, Godalming, UK) fitted with 35 mm cylindrical probe and 5 kg load cell. Samples were compressed twice at a distance of 20 mm, at speed of 0.5 mm/s and pre-test speed of 1.0 mm/s, in a test vessel that was 68 mm in diameter. Hardness (N), adhesiveness (N∙s), cohesiveness, springiness, and gumminess (N) were determined. Measurements were performed on starch pastes (5%) prepared with the assistance of Brabender viscograph (Section 3.7), immediately after preparation.

### 3.10. Statistical Analysis

The data are presented as mean values of three replicates ± standard deviation. One-way analysis of variance (ANOVA) and Tukey’s post hoc test were performed to determine statistically homogenous subsets at α = 0.05. Principal component analysis (PCA) was performed based on the correlation matrix. Cluster analysis (CA) was conducted based on Ward’s method, and Euclidean distance was used as a measure of similarity. The statistical analyses were performed using Statistica 13.3 (TIBCO Software Inc., Palo Alto, CA, USA).

## 4. Conclusions

Starch preparations derived from waxy plant mutants are characterized only by residual amounts of amylose that is accompanied by a lower phosphorus content (compared to their normal counterparts). Other microconstituents of the starch granule are not affected by this phenomenon. The morphology of starch granules is also similar, with the only exception observed for corn starch, which consists also of larger granules, resulting in a larger granule size span. As a result of being constituted almost entirely of amylopectin, the conformation of waxy starch macromolecules in solution is more open, as indicated by a higher hydrodynamic radius and a lower radius of gyration. The presence of amylose confers the ability to coagulate starch sol into gel, resulting in substantial changes to the rheological properties of starch paste, in particular, pronounced consistency index, non-Newtonian flow behavior, thixotropy, and elastic properties. On the other hand, waxy starch pastes are characterized by a more viscous flow characteristic and a smoother texture. Finally, the gelatinization temperature of waxy starch is significantly lower than that of normal preparations.

Investigated preparations of potato, corn, and rice starch varieties were also characterized by significantly different molecular and rheological properties due to their botanical origin. The greatest differentiation was observed between tuber and cereal starches. The observed differences were more notable for normal than waxy preparations. The pasting, rheological, and texture characteristics of the investigated starches suggest different fields of applications in food technology for these products. Nonetheless, both normal and waxy potato starch preparations were characterized by the most unique physicochemical properties.

Recommended specific areas of use of native waxy starch preparations may include food applications in which acetylated and oxidized starch are used. In that regard, particular attention should be paid to waxy potato and also, to some extent, waxy corn starch. The use of rice starches might be limited to a specific niche, such as application in granular form, in cases where very small granules are desired (e.g., cosmetics).

## Figures and Tables

**Table 1:** Proximate composition of starch granules and paste clarity.

Starch	Amylose %	Protein %	Ash %	Phosphorus %	Paste Clarity %T
Potato	19.3 ± 0.7 ^c^	0.06 ± 0.02 ^a^	0.33 ± 0.01 ^c^	0.08 ± 0.00 ^f^	80.1 ± 0.1 ^e^
Waxy potato	3.0 ± 0.5 ^a^	0.07 ± 0.03 ^a^	0.27 ± 0.02 ^c^	0.06 ± 0.00 ^e^	86.0 ± 0.5 ^f^
Corn	26.1 ± 0.5 ^d^	0.33 ± 0.07 ^b^	0.10 ± 0.02 ^a^	0.02 ± 0.00 ^c^	2.1 ± 0.1 ^a^
Waxy corn	2.5 ± 0.4 ^a^	0.14 ± 0.02 ^a^	0.08 ± 0.01 ^a^	0.00 ± 0.00 ^a^	48.6 ± 0.4 ^d^
Rice	14.4 ± 0.6 ^b^	0.28 ± 0.05 ^b^	0.29 ± 0.01 ^c^	0.03 ± 0.00 ^d^	3.9 ± 0.1 ^b^
Waxy rice	2.9 ± 0.2 ^a^	0.75 ± 0.03 ^c^	0.17 ± 0.01 ^b^	0.01 ± 0.00 ^b^	7.9 ± 0.1 ^c^

Values marked with the same letter do not differ significantly *p* > 0.05.

**Table 2:** Starch granule size distribution.

Starch	D1 µm	D5 (Median) µm	D9 µm	Span -
Potato	16.8 ± 0.4 ^d^	34.5 ± 0.1 ^e^	58.5 ± 1.7 ^e^	1.21 ± 0.06 ^b^
Waxy potato	16.7 ± 0.2 ^d^	33.2 ± 0.7 ^d^	52.9 ± 1.2 ^d^	1.09 ± 0.01 ^b^
Corn	8.4 ± 0.2 ^b^	13.2 ± 0.3 ^b^	20.3 ± 0.7 ^b^	0.90 ± 0.02 ^a^
Waxy corn	9.8 ± 0.2 ^c^	20.3 ± 0.03 ^c^	39.9 ± 1.9 ^c^	1.48 ± 0.10 ^c^
Rice	1.0 ± 0.0 ^a^	4.8 ± 0.1 ^a^	8.6 ± 0.2 ^a^	1.59 ± 0.02 ^c^
Waxy rice	1.1 ± 0.0 ^a^	5.5 ± 0.1 ^a^	9.8 ± 0.3 ^a^	1.58 ± 0.06 ^c^

Values marked with the same letter do not differ significantly *p* > 0.05.

**Table 3:** Hydrodynamic parameters of native starch macromolecules.

**Starch**	**M_w_/M_n_** **(-)**	**IV** **(dl/g)**	**R_h_** **(nm)**	**R_g_** **(nm)**	**R_g_/R_h_** **(-)**	**M-H α** **(-)**	**Branches** **(-)**
Potato	17.45 ± 1.04 ^c^	1.36 ± 0.02 ^d^	77 ± 3 ^a^	153 ± 6 ^b^	2.00 ± 0.12 ^c^	0.305 ± 0.019 ^a^	7 ± 1 ^a^
Waxy potato	1.89 ± 0.18 ^a^	1.39 ± 0.02 ^d^	87 ± 0 ^bc^	105 ± 6 ^a^	1.21 ± 0.07 ^a^	0.298 ± 0.009 ^a^	8 ± 0 ^a^
Corn	1.80 ± 0.15 ^a^	0.72 ± 0.05 ^c^	92 ± 4 ^c^	193 ± 12 ^c^	2.10 ± 0.10 ^c^	0.298 ± 0.015 ^a^	24 ± 1 ^b^
Waxy corn	2.25 ± 0.21 ^a^	0.53 ± 0.03 ^a^	94 ± 1 ^c^	150 ± 7 ^b^	1.59 ± 0.06 ^b^	0.502 ± 0.040 ^b^	34 ± 5 ^c^
Rice	5.31 ± 0.47 ^b^	0.56 ± 0.03 ^ab^	81 ± 4 ^ab^	201 ± 15 ^c^	2.50 ± 0.27 ^d^	0.355 ± 0.012 ^a^	30 ± 2 ^b^
Waxy rice	1.99 ± 0.17 ^a^	0.64 ± 0.03 ^b^	106 ± 3 ^d^	155 ± 3 ^b^	1.47 ± 0.06 ^ab^	0.447 ± 0.068 ^b^	27 ± 4 ^b^

Values marked with the same letter do not differ significantly. Mw/Mn—polydispersity index, IV—intrinsic viscosity, Rh—hydrodynamic radius, Rg—radius of gyration, M-H α—Mark–Houwink equation α parameter, Branches—expressed as value per molecule.

**Table 4:** Parameters of Ostwald–de Waele equation and thixotropy of 5% starch pastes.

Starch	K (Pa∙s^n^)		*n* (-)		R^2^		Thixotropy (Pa∙s^−1^)
	0–600 s^−1^	600–0 s^−1^	0–600 s^−1^	600–0 s^−1^	0–600 s^−1^	600–0 s^−1^	
Potato	31.86 ± 0.98 ^c^	12.67 ± 0.12 ^e^	0.468 ± 0.001 ^bc^	0.592 ± 0.003 ^e^	0.976	0.999	49,535 ± 2496 ^c^
Waxy potato	10.93 ± 0.61 ^ab^	8.70 ± 0.32 ^d^	0.495 ± 0.006 ^c^	0.517 ± 0.004 ^bc^	0.987	0.998	10,054 ± 547 ^ab^
Corn	13.11 ± 5.09 ^b^	5.62 ± 1.33 ^c^	0.387 ± 0.044 ^a^	0.512 ± 0.024 ^ab^	0.991	0.996	7208 ± 3298 ^a^
Waxy corn	5.47 ± 0.36 ^a^	4.04 ± 0.06 ^ab^	0.508 ± 0.006 ^c^	0.543 ± 0.001 ^cd^	0.986	0.999	5503 ± 839 ^a^
Rice	5.93 ± 0.11 ^a^	2.65 ± 0.03 ^a^	0.439 ± 0.003 ^b^	0.554 ± 0.002 ^d^	0.998	0.998	5748 ± 58 ^a^
Waxy rice	7.84 ± 0.11 ^ab^	5.48 ± 0.01 ^bc^	0.439 ± 0.002 ^b^	0.486 ± 0.001 ^a^	0.995	0.999	13,515 ± 375 ^b^

Values marked with the same letter do not differ significantly *p* > 0.05. K—consistency index, *n*—flow behavior index (value indicating convergence with Newtonian flow).

**Table 5:** Power law equation constants for mechanical spectra of starch pastes.

Starch	Storage Modulus (G′)			Loss Modulus (G″)			Average Phase Angle Tg (δ)
	K′ (Pa/s^n^)	*n*′ (-)	R^2^	K″ (Pa/s^n^)	*n*″ (-)	R^2^	
Potato	38.0 ± 0.6 ^c^	0.106 ± 0.002 ^b^	0.969	7.71 ± 0.13 ^d^	0.232 ± 0.005 ^b^	0.983	0.202 ± 0.038 ^ab^
Waxy potato	4.1 ± 0.1 ^a^	0.338 ± 0.009 ^d^	0.999	2.48 ± 0.05 ^b^	0.393 ± 0.012 ^e^	0.999	0.607 ± 0.039 ^c^
Corn	339.3 ± 9.0 ^d^	0.042 ± 0.010 ^a^	0.998	19.31 ± 0.64 ^e^	0.150 ± 0.005 ^a^	0.979	0.057 ± 0.010 ^a^
Waxy corn	2.3 ± 0.1 ^a^	0.338 ± 0.001 ^d^	0.999	1.42 ± 0.04 ^a^	0.461 ± 0.008 ^f^	0.999	0.623 ± 0.101 ^c^
Rice	26.3 ± 0.6 ^b^	0.111 ± 0.001 ^b^	0.999	4.49 ± 0.09 ^c^	0.290 ± 0.009 ^c^	0.995	0.176 ± 0.041 ^ab^
Waxy rice	9.4 ± 0.2 ^a^	0.172 ± 0.003 ^c^	0.999	2.62 ± 0.07 ^b^	0.360 ± 0.009 ^d^	0.999	0.284 ± 0.073 ^b^

Values marked with the same letter do not differ significantly *p* > 0.05.

**Table 6:** Universal texture profile of 5% starch pastes.

Starch	Hardness N	Adhesiveness N∙s	Cohesiveness	Springiness	Gumminess N
Potato	0.42 ± 0.03 ^b^	0.21 ± 0.06 ^b^	0.74 ± 0.01 ^ab^	0.99 ± 0.01 ^a^	0.31 ± 0.02 ^a^
Waxy potato	0.35 ± 0.00 ^a^	0.00 ± 0.01 ^a^	0.73 ± 0.00 ^a^	1.00 ± 0.00 ^a^	0.26 ± 0.00 ^a^
Corn	0.57 ± 0.07 ^c^	0.92 ± 0.13 ^c^	0.78 ± 0.04 ^b^	0.96 ± 0.04 ^a^	0.44 ± 0.07 ^b^
Waxy corn	0.34 ± 0.00 ^a^	0.00 ± 0.00 ^a^	0.74 ± 0.00 ^ab^	1.00 ± 0.00 ^a^	0.25 ± 0.00 ^a^
Rice	0.37 ± 0.01 ^a^	0.02 ± 0.01 ^a^	0.75 ± 0.01 ^ab^	0.98 ± 0.02 ^a^	0.28 ± 0.01 ^a^
Waxy rice	0.38 ± 0.01 ^a^	0.01 ± 0.00 ^a^	0.73 ± 0.00 ^a^	1.02 ± 0.02 ^a^	0.28 ± 0.01 ^a^

Values marked with the same letter do not differ significantly *p* > 0.05.

## Data Availability

All data generated or analyzed during this study are included in this published article.
